# Phenotypic and Genetic Analyses of the *Varroa* Sensitive Hygienic Trait in Russian Honey Bee (Hymenoptera: Apidae) Colonies

**DOI:** 10.1371/journal.pone.0116672

**Published:** 2015-04-24

**Authors:** Maria J. Kirrane, Lilia I. de Guzman, Beth Holloway, Amanda M. Frake, Thomas E. Rinderer, Pádraig M. Whelan

**Affiliations:** 1 School of Biological, Earth and Environmental Sciences (BEES), University College Cork, Cork, Ireland; 2 Environmental Research Institute (ERI), University College Cork, Lee Road, Cork, Ireland; 3 USDA-ARS, Honey Bee Breeding, Genetics and Physiology Laboratory, 1157 Ben Hur Road, Baton Rouge, Louisiana 70820, United States of America; 4 USDA-ARS, Honey Bee Breeding, Genetics and Physiology Laboratory, 1157 Ben Hur Road, Baton Rouge, Louisiana 70820, United States of America; Universidade de São paulo, BRAZIL

## Abstract

*Varroa destructor *continues to threaten colonies of European honey bees. General hygiene, and more specific *Varroa* Sensitive Hygiene (VSH), provide resistance towards the *Varroa* mite in a number of stocks. In this study, 32 Russian (RHB) and 14 Italian honey bee colonies were assessed for the VSH trait using two different assays. Firstly, colonies were assessed using the standard VSH behavioural assay of the change in infestation of a highly infested donor comb after a one-week exposure. Secondly, the same colonies were assessed using an “actual brood removal assay” that measured the removal of brood in a section created within the donor combs as a potential alternative measure of hygiene towards *Varroa-*infested brood. All colonies were then analysed for the recently discovered VSH quantitative trait locus (QTL) to determine whether the genetic mechanisms were similar across different stocks. Based on the two assays, RHB colonies were consistently more hygienic toward *Varroa*-infested brood than Italian honey bee colonies. The actual number of brood cells removed in the defined section was negatively correlated with the *Varroa *infestations of the colonies (r^2^ = 0.25). Only two (percentages of brood removed and reproductive foundress *Varroa*) out of nine phenotypic parameters showed significant associations with genotype distributions. However, the allele associated with each parameter was the opposite of that determined by VSH mapping. In this study, RHB colonies showed high levels of hygienic behaviour towards *Varroa -*infested brood. The genetic mechanisms are similar to those of the VSH stock, though the opposite allele associates in RHB, indicating a stable recombination event before the selection of the VSH stock. The measurement of brood removal is a simple, reliable alternative method of measuring hygienic behaviour towards *Varroa* mites, at least in RHB stock.

## Introduction

Hygienic behaviour in honey bees was first studied by Rothenbuhler [1] in relation to the brood disease American Foulbrood (AFB). Hygiene involves three components, namely: the detection, uncapping and removal of diseased or dead brood from the hive [2]. In addition to AFB, it is a means of resistance to a number of in-hive pests including wax moths [3] and small hive beetles [4].

The invasive mite *Varroa destructor* Anderson and Trueman (Mesostigmata: Varroidae) continues to be regarded as the greatest threat facing honey bees worldwide [5]. In the mite’s native host, *Apis cerana*, *Varroa* only reproduces in drone brood, ensuring populations remain at levels that do not result in significant harm (Rath, 1999). In addition, hygienic removal of *Varroa*-infested brood contributes to resistance in this bee [6, 7]. Hygienic behaviour has been studied widely in *A*. *mellifera* and has been found to be one of a number of traits that are capable of instilling resistance towards the *Varroa* mite in its invaded host [2, 8]. Hygienic behaviour is a heritable trait [9, 10] and *Varroa* specific hygiene is the basis of resistance in the *Varroa* Sensitive Hygienic (VSH) stock from the USDA Laboratory in Baton Rouge, LA [11]. General hygienic behaviour also confers some resistance toward the *Varroa* mite in the Minnesota hygienic line [11, 12] and may contribute to the resistance of *Varroa*-surviving colonies in France [13].

A large proportion of *Varroa* mites in VSH colonies enter cells and either die, produce no progeny, produce only male or only immature progeny [10]. In 2006, Ibrahim and Spivak [14] found that non-reproduction of mites was related to hygienic behaviour and the term *Varroa* Sensitive Hygiene (VSH) was coined [15]. These bees preferentially remove mite-infested pupae that are between 3 to 5 days post-capping [15]. *Varroa* mite reproduction is heavily synchronized with the reproductive program of its bee host [16]. Foundress mites must produce at least a mature male and mature female mite within the natal cell in order to produce viable offspring [17]. Mites removed from cells by hygienic behaviour may be forced to reinvade new cells in order to complete reproduction [15]. A break in synchrony between the reproductive cycle of the re-invading mite and the development of its host has previously been shown to negatively impact mite reproduction [18, 19]. Thus, the removal behaviour of VSH stock significantly lowers the population of *Varroa* mites in these colonies.

Another *Varroa*-resistant stock maintained by the USDA is the Russian honey bee (RHB) [20]. RHB colonies maintain consistently lower levels of *Varroa* compared with unselected stocks [21–23]. No overwhelming resistance mechanism has been identified [23]. However, an increased phoretic period of *Varroa* on adult bees leads to mites being more susceptible to the bee’s grooming behaviour [24]. Using the freeze-killed brood (FKB) assay, [25] showed that RHB colonies consistently removed dead brood at levels high enough to be considered hygienic (>95% brood removed within 48 h over two assays). Non-reproduction of *Varroa* foundresses has also been found to be a contributing factor in the resistance of RHB stocks [26], [23]. This is in part due to the comb built by RHBs but could also be caused, in part, by RHB brood stimulating hygienic behaviour in adult bees [23].

Although originally thought to be controlled by simple Mendelian genetics [1], more recent studies have determined hygienic behaviour to be a more quantitative trait [27, 28] and in fact up to seven loci may be responsible for its expression [29, 30]. Quantitative trait loci (QTL) mapping of genotypes of individual VSH stock that uncapped and removed infested pupae identified candidate genes associated with vision, olfaction, memory and dopamine reception [31]. Those bees that were homozygous for the alleles identified as being associated with the trait were more likely to be observed expressing the behaviour [31]. Differential up-regulation and down-regulation of genes associated with neuronal wiring, olfaction and visual signalling has been shown in VSH and non-VSH stock [32]. In addition, VSH stock and Africanized bee genomic profiles were found to be similar indicating an overlap in the genetic mechanisms across the stocks [32]. It does not appear to be the scent of the mite itself that stimulates hygiene toward *Varroa*-infested brood [33]. Rather, hygienic bees preferentially detect and remove brood infested with DWV- transmitting mites, triggered by “deviant” volatile compounds released by the damaged infested pupae [34].

Hygienic behaviour towards *Varroa*-infested brood is a highly desirable trait for bee breeders and beekeepers to incorporate in their breeding programs. However, methods used to measure this trait can be both time-consuming and lacking in precision. Hygienic behaviour is measured using the pin-prick or freeze-killed brood (FKB) assay while VSH activity is generally measured by calculating the change (= reduction) in infestation of a highly infested donor brood frame placed into a test hive for one week. Many researchers have recognized the need for more simplified methods of measuring hygiene towards *Varroa-*infested brood [28, 35, 36]. Attempts have been made to correlate the number of uncapped cells with VSH activity but have proven unreliable [35]. In Europe, researchers favour the use of the pin-killed brood assay for measuring hygienic behaviour [13]. However, the removal of dead brood does not always correlate well with removal of *Varroa-*infested brood [37]. Improved understanding of the mechanisms underlying hygienic behaviour toward *Varroa*, as well as improved measurement techniques could lead to more successful breeding programs worldwide.

The main objective of this study was to determine whether the VSH trait was present in RHB colonies. Through genetic analysis, we investigated the association of the QTL identified by [31] with VSH stock in both RHB and Italian stocks. We also determined whether brood removal in a small section of brood was associated with both VSH activity and mite infestation, which could provide a simpler and more reliable screening method for hygienic brood removal.

## Materials and Methods

### Colony set up

Experiments were carried out at the USDA-ARS, Honey Bee Breeding, Genetics and Physiology Laboratory in Baton Rouge, Louisiana during August and September of 2013. Forty six colonies (Italian = 14, Russian = 32) were established in Spring of 2013. Italian honey bee queens were purchased from a commercial queen producer in California that advertises Italian honey bees while RHB queen lines were obtained from members of the Russian Honeybee Breeders Association (RHBA). All experiments were carried out at least two months after the introduction of queens, and only using colonies that had not superseded, in order to ensure that all bees were from the test queens.

### Phenotypic determination of VSH activity

In August 2013, all colonies were measured for the percentage of *Varroa*-infested brood removed (referred to as VSH activity in this study) using the “change in mite infestation” method of [37]. Briefly, a frame of *Varroa*-infested brood (sealed larvae, prepupae or white-eyed pupae) was taken from a highly infested donor colony and introduced into a test colony. Before introduction, the level of infestation of the test frame was determined by examining 200 brood cells, 100 on each side of the brood frame. The test frame was then inserted into the centre of the brood nest of a test colony. After 6–7 days post introduction, the frame was removed and infestation of the same cohort of brood was determined by pulling 200 cells of purple-eyed to tan-bodied pupae. The examination of 200 brood cells per colony has been the standard procedure for determining brood infestation in our earlier studies [24, 26, 28, 37] and has been adopted as the optimum number to determine the brood infestation of a colony by [38]. By measuring the infestation level of the same cohort of capped brood before and after introduction into the test colony, we can conclude that a reduction in infestation is related to hygienic removal of infested brood [37]. Test brood frames were obtained from 14 donor colonies separate from the test colonies. Only brood frames with 10–33% (average = 17.7% ± 5.6) infestations and with ≥50% brood area were used as donor frames. The VSH activity of the colony was determined using the equation:

$$\text{\% infested brood removed = }\frac{\left(\text{Initial infestation }-\text{ Final infestation}\right)}{\text{Initial infestation}}\;\times \text{ 100}$$

### Relationship between brood removal in a small test section and VSH activity (= change in infestation) of the test brood frame

**Fig 1:**
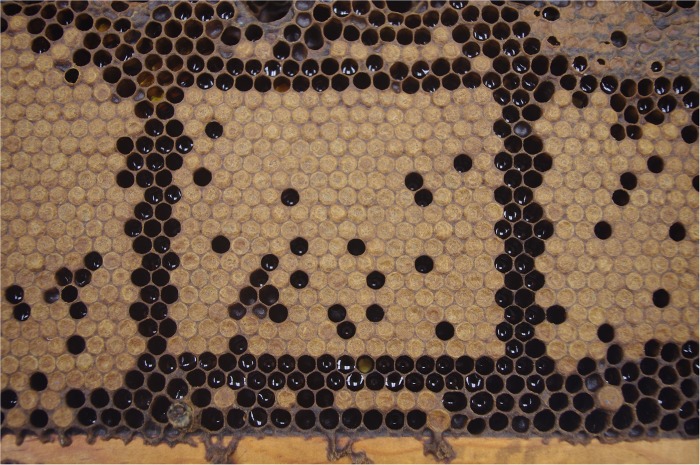
A small brood section of 238 cells created within an infested donor comb.

In order to determine whether or not brood removal was related to VSH activity, a small section of capped brood (average = 162 ± 35 cells) was delimited on each donor frame (used in the VSH assay) by removing the brood surrounding the section (Fig 1). The section was photographed before the donor frame was placed into the test colony and again after six to seven days, when VSH activity and other infestation parameters were also measured. By comparing both photographs the number of capped brood that had been removed, or cells that had been opened, by the bees during the test period was determined. While most brood had been completely removed, some remained open (possibly in the process of being removed), while others were recapped. The numbers of removed and opened brood were combined, which provided an overall measurement of total brood manipulation. The initial and final infestations of each brood section were also measured and VSH activity determined using the above equation.

### Assessment of parameters associated with hygiene in resident brood of test colonies

For each test colony, *Varroa* infestation, mite reproduction and cell recapping rates of resident brood were also determined. These parameters were measured by examining brood cells from 2–3 resident frames that contained purple-eyed pupae and older. The introduction of infested brood frames to measure VSH activity would have resulted in an increased infestation of all colonies. Measuring the natural infestations of resident brood frames, after mite introduction through donor combs, further allowed us to compare *Varroa* susceptibility in the two stocks. In addition, comparing the natural mite reproduction in un-manipulated resident brood frames enabled us to determine whether or not VSH behaviour lowers reproductive output in naturally infested cells, as suggested by our earlier study [18]. Brood cells were examined until 30 infested cells were obtained or, for colonies with lower levels of infestation, at least 1000 cells had been examined. A foundress *Varroa* was considered non-reproductive when no progeny was produced. Varroa families were examined based on our previous studies [24, 26] and according to the standard methods [38]. Average numbers of progeny per fertile foundress and average mite load per infested cell were also determined. Recapping prevalence of naturally infested cells was determined by carefully pulling back the capping of each cell on the resident brood frames; recapped brood cells lack a silk cocoon [39]. This recapping activity indicates that the bees opened the infested cells but these cells were subsequently recapped without removing the brood. The silk cocoon was either missing from the entire (complete recapping) or part of the inner surface (partial recapping).

### Genetic determination of VSH activity

From each of the 46 colonies assessed for the VSH trait, 96 pupae were collected and analysed for the nature of a single nucleotide polymorphism (SNP) marker associated with VSH. DNA extraction was performed according to established protocols [40]. Briefly, each individual pupa was ground between steel beads in 6M NaCl, 1% SDS by TissueLyser (Qiagen, LaJolla, CA USA), centrifuged, and the supernatant collected. DNA was pelleted from the supernatant using isopropanol and washed with 70% ethanol. DNA was resuspended in deionized water and subjected to SNP genotyping to assess the allele frequency per colony. The honey bee SNP 9–9224292 was previously identified by quantitative trait locus (QTL) mapping as strongly associated with uncapping and removing behaviours that are components of the VSH trait [31]. High throughput genotyping for this specific SNP was achieved by a PCR/restriction digest diagnostic test. The derived cleaved amplified polymorphism (dCAPs; http://helix.wustl.edu/dcaps/dcaps) PCR primers For -5’-CGCGTGTATGTGTGTATTTACAAAGTTCGG and Rev-5’-TACTTGCTCGTCCATCGTCCATA amplified a fragment containing the associated honey bee SNP. The forward primer contained an engineered mutation (cystine base at position 28 of 30) that worked in concert with the honey bee SNP (adenine or guanine) to generate a restriction site. Following standard PCR amplification, digestion of the product with Hpy188i (New England Biolabs, Beverly, MA, USA) and gel electrophoresis procedures, bands of 207 bp (guanine at SNP; hygienic allele as determined by QTL mapping) or 178 bp (adenine at SNP; non-hygienic as determined by QTL mapping) were distinguishable. The percentages of individual pupae exhibiting homozygous alleles were tabulated for each test colony. For each of the measured parameters, colony phenotypes were sorted to highlight the extreme 25% quartiles while excluding those phenotypes that lay within the middle 50%.

### Statistical Analyses

Prior to analyses, all data were checked for normality. Percentage data were not normal and thus were transformed using arcsine square root transformation to better approximate normality. Data on the number of progeny and mite load were normal. Untransformed means were presented. Data were analysed for outliers and one outlier was deleted before analyses. Student’s t-tests were performed to determine significance between the extremes of colony phenotypes and genotypes. Differences between stocks for measurements related to hygiene were also determined using student’s t-tests. Pearson’s product moment correlation was carried out on each of the variables measured with VSH activity to determine an association. This was performed using the whole data set as well as on RHB and Italian stocks separately. Statistics were carried out using the software package R [41].

## Results

### Phenotypic determination of VSH activity

**Fig 2:**
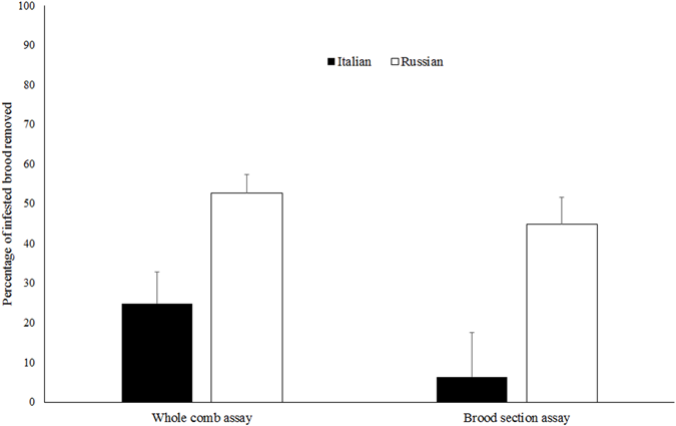
A comparison of the percentages of *Varroa*-infested brood removed by Italian and Russian honey bees using the whole comb and brood section assays. Percentage of *Varroa*-infested brood removed was calculated as the % change in *Varroa* infestation of donor combs.

**Fig 3:**
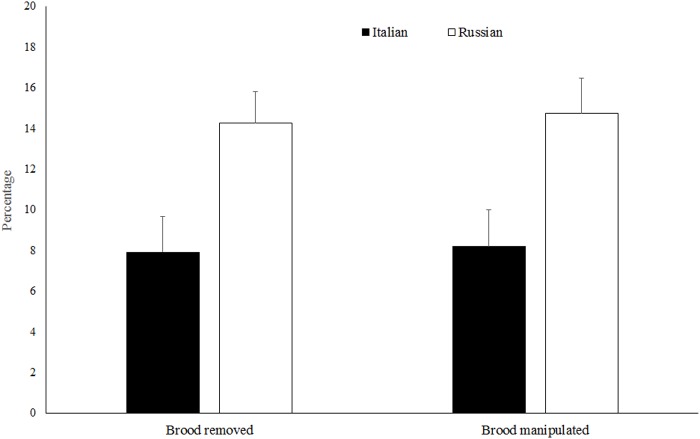
Percentages of brood removed and manipulated by Italian and Russian honey bee colonies in test brood sections of donor combs.

RHB colonies were significantly more hygienic toward *Varroa*-infested brood than Italian colonies and this was true using both the whole donor comb (*t* = 2.91, d.f. = 42, *P* < 0.01) and the small section of brood created within the donor comb (*t* = 2.75, d.f. = 42, *P <* 0.01) (Fig 2). Both brood removal (*t* = 2.15, d.f. = 39, *P* < 0.05) and total manipulated brood (*t* = 2.25, d.f. = 39, *P* < 0.05) were also significantly higher in the RHB than in the Italian colonies (Fig 3). These two measurements were positively correlated with the levels of VSH activity using both the whole comb and the brood section methods (Table 1). At the end of the experiment, reproductive success of foundress *Varroa* (production of at least one progeny) in the donor combs was similar for both honey bee stocks (Italian = 79.23 ± 3.57%; RHB = 71.13 ± 3.3%) (*t* = 1.68, d.f. = 42, *P* = 0.102).

**Table 1:** Results of the regression analyses relating percentages of *Varroa*-infested brood removed for whole comb and brood section of donor frames, and brood removal of brood section to different measurements related to hygiene.

	Overall			Italian			Russian		
Measurement	R	r^2^	*P* value	r	r^2^	*P* value	r	r^2^	*P* value
A. % *Varroa*-infested brood removed (donor comb)									
% *Varroa*-infested brood removed (brood section of donor comb)	0.55	0.30	< 0.001	0.23	0.05	0.474	0.51	0.26	< 0.01
% brood removal (brood section of donor comb)	0.36	0.13	< 0.05	-0.17	0.03	0.586	0.45	0.20	< 0.05
% manipulated brood (brood section of donor comb)	0.39	0.15	< 0.05	-0.14	0.02	0.661	0.46	0.21	< 0.05
% infestation (resident brood)	-0.43	0.18	< 0.01	-0.27	0.07	0.414	-0.31	0.10	0.090
% of NR foundresses* (resident brood)	0.24	0.06	0.111	0.30	0.09	0.100	0.01	0.0001	0.960
% recapped cells (resident brood)	0.22	0.04	0.146	0.36	0.13	0.241	0.10	0.01	0.571
B. % *Varroa*-infested brood removed (brood section of donor comb)									
% brood removal (brood section of donor comb)	0.31	0.10	< 0.05	0.29	0.08	0.354	0.25	0.06	0.191
% manipulated brood (brood section of donor comb)	0.32	0.10	< 0.05	0.30	0.09	0.343	0.23	0.05	0.231
C. % brood removal (brood section of donor comb)									
% initial infestation (brood section of donor comb)	0.57	0.32	< 0.001	0.20	0.04	0.532	0.66	0.44	< 0.001
% infestation (resident brood)	-0.5	0.25	< 0.001	-0.18	0.03	0.558	-0.53	0.28	< 0.01
% of NR foundresses* (resident brood)	0.38	0.14	< 0.05	0.05	0.003	0.857	0.46	0.21	< 0.05
% recapped cells (resident brood)	0.35	0.12	< 0.05	0.28	0.08	0.376	0.28	0.08	0.131

Percentage of *Varroa*-infested brood removed was calculated as the % change in *Varroa* infestation of donor combs.

*NR = no progeny produced.

### Assessment of parameters associated with hygiene

*Varroa* infestation rates of resident brood were significantly lower in the RHB than in the Italian colonies (Table 2). This was evidenced by the higher number of cells (mean = 644 vs 348 cells) that were opened in order to obtain 30 infested cells per RHB colony. For the proportion of non-reproductive (NR) foundresses (no progeny), RHB colonies supported higher NR than Italian colonies (Italian = 4.36 ± 1.33, RHB = 13.11 ± 1.84, *P* < 0.010) (Table 1). NR was also correlated with actual brood removal in the test brood section (r^2^ = 0.10, *P* < 0.05) but not with VSH activity using the whole comb measurement (r^2^ = 0.06, *P* = 0.111) (Table 1). Recapping rates of infested brood cells were high for both stocks but significantly higher in RHB colonies (Italian = 64.36 ± 6.46, RHB = 77.81 ± 3.79, *P* < 0.001) (Table 2). Whether the brood cells were recapped or not, the average number of progeny per foundress and mite load per infested cell were higher in the resident brood combs of Italian compared to RHB colonies (Table 2). Interestingly, the average number of progeny and the average mite load were slightly higher in recapped compared with non-recapped cells for both stocks. *Varroa* infestation of colonies (resident combs) was negatively correlated with both brood removal (r^2^ = 0.25, *P* < 0.01) and the standard VSH activity (r^2^ = 0.18, *P* < 0.05). NR and recapping rates were not associated with VSH activity but both measurements were positively correlated with brood removal (NR: r^2^ = 0.14, *P* < 0.05, Recapping: r^2^ = 0.12, *P* < 0.05) (Table 1).

**Table 2:** Sample sizes, means (± SE) of different infestation parameters in resident brood of Italian and Russian honey bee colonies, and results of the student’s t-tests.

Parameters	Italian	Russian	*P*-values
Number of test colonies	14	32	
Number of cells examined	4,525	21,896	
Number of cells examined per colony	348 ± 76	644 ± 53	
% Infested cells	14.42 ± 3.10^a^	6.02 ± 1.14^b^	< 0.001
% Multiply infested brood	7.69 ± 2.40	3.57 ± 0.80	0.059^ns^
% NR foundresses	4.36 ± 1.33^b^	13.11 ± 1.84^a^	< 0.010
% Infested cells recapped	64.36 ± 6.46^b^	77.81 ± 3.79^a^	< 0.001
Number of progeny per fertile foundress			
Recapped	3.96 ± 0.11^a^	3.81 ± 0.08^b^	< 0.001
Not recapped	3.86 ± 0.15^a^	3.79 ± 0.20^b^	< 0.001
Overall	3.94 ± 0.12^a^	3.80 ± 0.07^b^	< 0.001
Mite Load			
Recapped	5.28 ± 0.25^a^	4.48 ± 0.13^b^	< 0.001
Not recapped	4.70 ± 0.21^a^	4.16 ± 0.23^b^	< 0.001
Overall	5.05 ± 0.16^a^	4.41 ± 0.11^b^	< 0.001

NR = no progeny. Rows with different letters are significantly different at *P* < 0.05.

ns = not significant.

### Genetic determination of VSH activity

Of the nine colony phenotypes measured, two parameters showed significant associations with genotype distributions: percentage of brood removed in the brood section and percentage of reproductive foundresses (produced at least one progeny) of the resident combs (Table 3). Interestingly, the allele associated with each parameter is the opposite of that determined by VSH mapping [31], wherein the 207 bp band segregated with the hygienic behaviours. In this study, the 207 bp band segregates with decreased brood removal rates and increased percentage of foundress *Varroa* mites that produced progeny, both of which are indicative of non-hygiene. The other seven parameters studied showed no statistically significant relationship with allele frequency (Table 3).

**Table 3:** *P*- value of associations between allele homozygosity and colony phenotypes.

Colony phenotypes	Homozygous 178 bp band	Homozygous 207 bp band
% *Varroa*-infested brood removed (donor comb)	0.641	0.905
% *Varroa*-infested brood removed (brood section)	0.740	0.828
% brood removal (brood section)	0.503	0.025**
% opened brood (brood section)	0.489	0.090
% manipulated brood * (brood section)	0.767	0.078
% reproductive foundress* (resident brood)	0.034**	0.144
% foundress with viable progeny* (resident brood)	0.597	0.451
% infestation (resident brood)	0.631	0.188
% recapped cells (resident brood)	0.290	0.340

Percentage of *Varroa*-infested brood removed was calculated as the % change in *Varroa* infestation of donor combs. Manipulated brood = brood removed + opened brood;

*reproductive foundress mites = produced one or more progeny; viable progeny = with adult male and young daughter.

**significant at *P* < 0.05.

## Discussion

This study confirmed that Russian honey bees express relatively high levels of hygiene towards *Varroa*-infested brood, similar to levels reported for the VSH stock [15, 37]. We also observed that the alleles associated with VSH [31] were associated with two of the measurements (percentage brood removal and percentage reproductive foundresses) assessed. However, these alleles did not show any significant association with VSH activity measured by the standard “change in infestation” assay of [37]. The molecular marker identified by [31] in the VSH QTL was determined by observing individual honey bees from a mapping population performing uncapping or removing tasks in a test arena. This association of the published VSH marker with standard colony level VSH activity has not yet been proven, only demonstrated in an artificial mapping population. It is possible, therefore, that the trait is not identical in RHB stocks and so the simplified measurement of VSH by [37] may not be the most appropriate method of phenotyping bees for the trait across all stocks. Instead, the actual removal of infested brood cells seems to better associate with the hygienic removal of *Varroa*-infested brood in RHB colonies. We presumed that the brood removed by the RHBs were infested since brood removal was highly correlated (r^2^ = 0.44) with the infestation of brood sections within the donor combs.

A brood removal trait is likely to be ubiquitous across honey bee stocks, serving as a mechanism to maintain brood and colony health. It is believed to be a multi-genic trait yet only a single strong QTL has been identified [28]. While the gene function conferring the trait remains unknown, the genetic responsibility may be similar across honey bee stocks. Indeed, we showed that a significant relationship exists between the QTL identified in VSH-selected Italian honey bee stock [31] and in RHB colonies. The most significant association was with brood removal, again indicating that the behavioral mechanism remains the same. The association of the alternative allele to the positive phenotypes in both RHB and commercial Italian stocks indicates that perhaps a stable chromosomal recombination event occurred in one stock of Italian honey bees prior to the selection of the VSH trait in the initial breeding program [15, 42]. While the recombination event is not unexpected [40], it does indicate that future marker-assisted selection (MAS) programs using associated markers rather than causative markers will require allele analysis to be sure that the appropriate variation of the trait is being selected.

Suppressed mite reproduction is thought to be a trait of VSH stock [14, 42]. In this study, we found significantly lower mite reproduction in RHB colonies than in unselected Italian colonies and this was associated with the previously identified hygienic QTL. Non-reproduction of mites has been shown to be higher in RHB colonies than in unselected Italians due, in part, to a comb effect [23]. The reproductive cycle of *Varroa* mites can be affected by hygienic removal and manipulation of cells that breaks the synchrony between the mite’s reproductive cycle that of its host [18]. High VSH activity and lower reproductive success of mites in Russian brood indicate that hygienic removal of infested brood in RHB colonies also contributes to the lower mite reproduction in these bees. Hygienic removal of *Varroa*-infested brood does not always lead to lower mite reproduction however. For example, no change in fertility of mites in colonies of MN hygienic bees crossed with VSH stock was detected [43]. Therefore, it has been argued that selection for removal of infested brood does not necessarily mean selection for suppressed mite reproduction. Rather oviposition ratio (number of pupae with ovipositing mites/number of pupae with non ovipositing mites) might be a better criterion to ensure both removal of infested cells and suppressed mite reproduction [36]. The fact that we did not find a relationship between VSH activity and NR supports this argument. However, we did find a correlation between the actual removal of brood in the test brood section and NR in resident brood of RHB colonies. This indicates that brood removal might be an alternative, reliable measurement of VSH activity in this stock. Nevertheless, since the correlation coefficient only accounted for about one fifth of the variation, it is best to validate the reliability of this brood removal assay using more colonies and more honey bee stocks.

No difference was detected between stocks in the number of mites that produced progeny after the week-long exposure of the donor brood frame. However, significant differences between the stocks were detected in the resident brood with RHB supporting higher NR than the Italian honey bees. This observation is in contrast to the findings of [35, 36, 42] who found lower reproduction of mites in the test comb after one week exposure to VSH stock. The high NR of *Varroa* in resident brood suggests that the effect of brood removal on mite reproduction is a delayed one. This delayed effect indicates that the removal of infested brood from donor combs impacted mite reproduction by breaking synchrony in the reproductive cycle of the exposed mite when re-invading resident brood [18]. Unfortunately, it was not possible for us to compare the reproductive ability of mites in recapped versus non-recapped cells as the numbers of non-recapped cells were low, particularly in RHB colonies. However, from the small numbers of non-recapped cells that we did observe in RHB colonies, reproduction in non-recapped cells was lower than in recapped cells. The higher mite load in recapped cells may also indicate invasion of mites when the cells were opened.

This present study confirms our earlier findings indicating that hygienic behaviour towards mite-infested brood reduces reproductive success of *Varroa* [18]. This trait can be identified by counting the number of brood removed, which is further supported by the association of the VSH QTL with our measure of removal and NR. In addition, brood removal had a stronger negative relationship with infestation of resident brood than the standard VSH measurement. This brood removal assay still requires the sourcing of infested donor comb but eliminates the need for a final evaluation of infestation or reproduction.

The highest positive correlation in this study was found between the initial infestation of the test brood section and the percentage of brood removed from the section. This finding indicates that the bees were responding to a threshold of infestation before they expressed the behaviour to any meaningful degree. There is an intrinsic fitness cost associated with removing young brood from a colony, so that the number of infested cells may need to reach a critical threshold before bees will begin to perform hygienic behaviour [44]. In other words, there should be a point at which the fitness costs associated with the level of infestation exceeds the cost associated with removing young bees from the population. At that point, the colony moves from tolerating the mite to actively resisting it [44]. The sensitivity of a colony to this level could be an important factor in resistance. It has previously been shown that detection and removal of *Varroa*-infested cells increases as colony infestation rises in RHB colonies, VSH stock and unselected “survivor” colonies [3]. Colonies bred for hygienic behaviour toward FKB are productive in commercial apiaries [12, 21] and their task threshold response could be a contributing factor in this. It has been found that the “cost” of hygienic removal of *Varroa*-infested cells is similar for resistant and susceptible stocks so that the difference in actual removal is probably due to differences in detection ability [44]. Indeed, through proboscis extension reflex conditioning, [45] showed that, in comparison to susceptible bees, hygienic bees resistant to Chalkbrood disease needed a lower stimulus to detect diseased brood. Alternatively, two stocks may differ in the level of mites tolerated by the colony; resistant stocks such as VSH and RHB having a lower behavioural threshold to mite density [15].

Rates of recapping were high for both RHB and Italian stocks, though significantly higher in RHB colonies. The combination of high infestations, low VSH activity and high levels of recapping in Italian honey bees suggests low expression of the genes associated with removal. In their 40 h test of VSH activity, [15] found no difference in the number of uncapped infested cells between unselected and selected VSH stocks. However, there was a significant difference in the reduction of infestation between the two groups of bees. Again, this observation indicates that the unselected Italian bees were uncapping the infested cells, but recapped the cells rather than remove the brood. Recapping was not related to VSH activity in this study but did show a correlation with brood removal. However, this correlation did not hold when the separate stocks were analysed indicating that it may be a weak correlation. This result supports the finding of [35] that recapping is not a useful measurement for VSH. High levels of recapping of infested brood at low levels of infestation, as was seen in resident RHB combs, can also be explained by task threshold responses. Individual workers in a colony vary in their behavioural thresholds for hygiene towards *Varroa*-infested pupae so that the most sensitive workers detect infested cells at lower infestations [27]. It is possible that less sensitive workers may then recap these cells before the brood has been removed [39]. Only at higher infestations would larger numbers of bees detect and remove the infested pupae [27].

There was a considerable degree of variation in the measurements of each variable which probably influenced the strength of the correlations. Sampling error has been attributed to the apparent gain in mite infestation seen in VSH testing where the authors reported 15 of the 61 test colonies showed apparent mite gain after a one-week exposure [37]. Using the whole comb measurements, only 2 out of 45 colonies displayed an increase in infestation in the present study. However, mite-gain was recorded in 8 of the 45 colonies with the brood section method. Nevertheless, considering the significant relationship between brood removal in the section and overall infestation of resident brood, we propose this method as a quick screening for hygienic behaviour. This method does not require the use of harsh chemicals required for the FKB assay but yet gives a better indication of *Varroa*-specific hygiene. [15] found a significant difference between the removal of mite infested pupae one to five days post-capping between unselected and selected VSH while removal of older pupae was similar between the two groups. A follow-up study to compare the actual removal of brood cells in an infested section up to 5 days post-capping with infestation might provide an even better screening method for hygienic behaviour.

In conclusion, RHB colonies express hygienic behaviour toward *Varroa-*infested brood at a rate similar to that of the VSH selected stock. The behaviour appears to be under the control of a similar QTL as the VSH stock. Interestingly, the opposite alleles associate with brood removal in RHB colonies, indicating a stable recombination event. This means that future marker assisted selection programs will require the use of causative markers or allele analysis to be effective. Observations on the removal of brood in a section of an infested donor comb after a one week of exposure can provide a rapid and reliable screening method for this behaviour. This method should be tested in other stocks to determine its usefulness in future breeding programs.

## Supporting Information

S1 Data Supporting data. (TXT)
